# The boron-oxygen core of borinate esters is responsible for the store-operated calcium entry potentiation ability

**DOI:** 10.1186/1471-2210-11-1

**Published:** 2011-01-26

**Authors:** Olivier Dellis, Pierre Mercier, Christine Chomienne

**Affiliations:** 1 INSERM UMR-S 940, Institut Universitaire d'Hématologie - Université Denis Diderot Paris 7, Hôpital Saint Louis, Paris, France; 2 INSERM UMR-S 757, Université Paris Sud 11, Orsay, France

## Abstract

**Background:**

Store-Operated Calcium Entry (SOCE) is the major Ca^2+ ^ion entry pathway in lymphocytes and is responsible of a severe combined immunodeficiency (SCID) when deficient. It has recently been observed or highlighted in other cell types such as myoblasts and neurons, suggesting a wider physiological role of this pathway. Whereas Orai1 protein is considered to be the channel allowing the SOCE in T cells, it is hypothesized that other proteins like TRPC could associate with Orai1 to form SOCE with different pharmacology and kinetics in other cell types. Unraveling SOCE cell functions requires specific effectors to be identified, just as dihydropyridines were crucial for the study of Ca^2+ ^voltage-gated channels, or spider/snake toxins for other ion channel classes. To identify novel SOCE effectors, we analyzed the effects of 2-aminoethyl diphenylborinate (2-APB) and its analogues. 2-APB is a molecule known to both potentiate and inhibit T cell SOCE, but it is also an effector of TRP channels and endoplasmic reticulum Ca^2+^-ATPase.

**Results:**

A structure-function analysis allowed to discover that the boron-oxygen core present in 2-APB and in the borinate ester analogues is absolutely required for the dual effects on SOCE. Indeed, a 2-APB analogue where the boron-oxygen core is replaced by a carbon-phosphorus core is devoid of potentiating capacity (while retaining inhibition capacity), highlighting the key role of the boron-oxygen core present in borinate esters for the potentiation function. However, dimesityl borinate ester, a 2-APB analogue with a terminal B-OH group showed an efficient inhibitory ability, without any potentiating capacity. The removal or addition of phenyl groups respectively decrease or increase the efficiency of the borinate esters to potentiate and inhibit the SOCE. mRNA expression revealed that Jurkat T cells mainly expressed Orai1, and were the more sensitive to 2-APB modulation of SOCE.

**Conclusions:**

This study allows the discovery of new boron-oxygen core containing compounds with the same ability as 2-APB to both potentiate and inhibit the SOCE of different leukocyte cell lines. These compounds could represent new tools to characterize the different types of SOCE and the first step in the development of new immunomodulators.

## Background

In lymphocytes, after T or B cell receptor cross-linking, inositol 1,4,5-trisphosphate (IP_3_) is synthesized [1] and induces Ca^2+ ^ion release from the lumen of the endoplasmic reticulum (ER), allowing the opening of Ca^2+ ^selective plasma membrane channels, known as store-operated channels (SOC). The resulting increase of the intracellular calcium concentration ([Ca^2+^]_i_) allows activation of NFAT (Nuclear Factor of Activated T cells) [2]. Inhibition of this Store-operated Calcium Entry (SOCE) by SKF96365 impairs T lymphocyte activation and subsequently interleukin 2 synthesis [3]. Recent studies have described two proteins playing key roles in SOCE: STIM1 and Orai1. STIM1, present in the ER membrane, senses the luminal Ca^2+ ^concentration, and translocates near the plasma membrane during Ca^2+ ^release, where it directly interacts with Orai1 protein forming the channel pore [4-6]. The R91W mutation of Orai1 renders the channel non-functional, and is responsible of a severe immunodeficiency [4]. Two Orai1 homologue genes have been described, Orai2 and Orai3. When expressed in HEK293 cells (with STIM1), the three Orai are able to produce or increase the SOCE [7]. However, despite their high homology, only Orai1 is able to restore the SOCE of SCID T cells [4,8]. Furthermore, Orai2 and Orai3 could show slight differences in kinetics and pharmacology.

Due to the key role of Ca^2+ ^influx in lymphocyte activation and proliferation, the use of effectors to modulate the Orai1-containing channels has appeared as a new and promising way to modulate lymphocyte activities and could represent a new way for the treatment of inflammatory diseases [9,10]. Although several pharmaceutical companies have developed molecules acting on SOCE, no specific SOCE effectors have been characterized [11].

One of the most interesting and promising molecule is a boron-containing molecule, 2-aminoethyl diphenylborinate (2-APB). 2-APB was originally described as a plasma membrane permeant inhibitor of IP_3 _receptors in human platelets and neutrophils [12], however it also impairs Sarcoplasmic-Endoplasmic Reticulum Ca^2+ ^ATPase activity at high concentrations (Kd > 200 μM), inducing a store Ca^2+ ^leak [13-15] and directly blocks SOCE in the same range of concentration as for IP_3 _receptor inhibition [16]. Furthermore, 2-APB has a dual effect on Jurkat T cell SOCE: potentiation at low concentration (1-5 μM) and inhibition at > 50 μM [17]. Similar behaviour has been described on human T, rat basophilic leukemic RBL-2H3 and chicken B DT40 cells [4,17,18]. 2-APB is also able to activate members of the TRP channel family at high concentrations (100 μM, TRPV1, V2 and V3 [19,20]) and to inhibit some others (TRPC3, C6 and C7 [21]). The effects of 2-APB on SOCE has been extensively studied in T cells but data on other cell types of hematopoietic origin is poorly documented.

**Figure 1:**
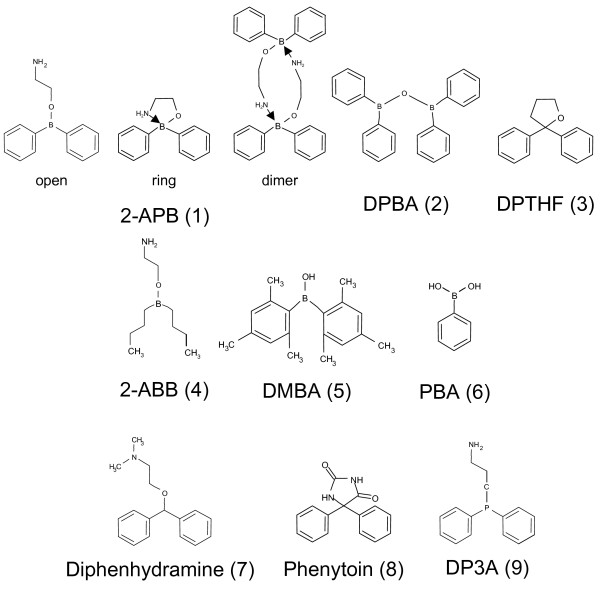
**Structure of 2-APB and analogues**. Three structures of 2-APB are depicted besides analogues: open-chain monomer, ring monomer when a ring is created by an N → B coordinate bond, dimeric form, when N → B bonds are created between two molecules of 2-APB.

**Figure 2:**
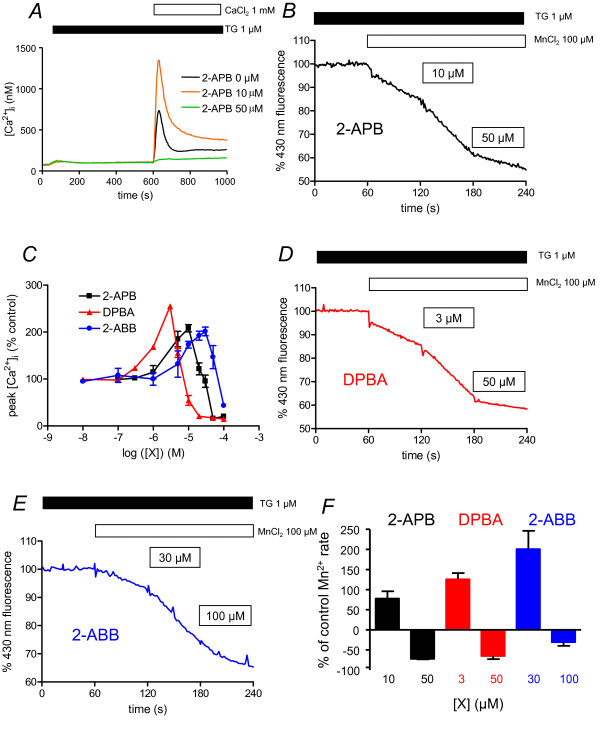
**Borinate esters have dual effects on store-operated calcium entry**. A. Cytosolic calcium concentration measurement of BL41 cells using indo-1 fluorescence. Cells were treated 10 min with TG 1 μM to allow Ca^2+ ^release from ER and opening of SOC channels. After 10 min, 1 mM CaCl_2 _was re-added, allowing Ca^2+ ^entry through SOC channels. Different 2-APB concentrations were applied 30 s prior to CaCl_2_. Error bars were omitted for clarity (maximum ~ ± 70 nM). B. Mn^2+ ^quenching of indo-1 in BL41 cells stimulated by potentiating and inhibitory concentrations of 2-APB. Cells were pre-treated 10 min with TG 1 μM allowing Ca^2+ ^release from ER and opening of SOC channels. 60 s after beginning of the recordings, 100 μM MnCl_2 _was added. At 120 s, potentiating concentration of 2-APB (10 μM) was added, followed 60 s later by inhibiting concentration of 50 μM. Fluorescence of indo-1 was recorded at 430 nm. Results expressed relative fluorescence prior to MnCl_2 _adding. C. 2-APB, DPBA and 2-ABB concentrations differently regulate SOCE of BL-41 cells. Experiments were done as in figure 1A, and peak [Ca^2+^]_i _was expressed as % of peak [Ca^2+^]_i _recorded in absence of any borinate ester compounds. Borinate ester compounds were added 30 s prior to CaCl_2_. D. Mn^2+ ^quenching of indo-1 in BL41 cells stimulated by potentiating and inhibitory concentrations of DPBA. Same as in figure 2B, except that 3 and 50 μM DPBA were used. E. Mn^2+ ^quenching of indo-1 in BL41 cells stimulated by potentiating and inhibitory concentrations of 2-ABB. Same as in figure 2B, except that 30 and 100 μM 2-ABB were used. F. Values of Indo-1 quenching in presence of the borinate esters were normalised to values obtained in the lonely presence of Mn^2+ ^ions.

Recently, several works have been published on 2-APB analogues. Thus, several analogues of 2-APB (**1**; Figure 1) have been described and tested on platelets and CHO cells, where 2-APB and analogues are only inhibitory [22-24]. On these cells, the boron-oxygen core (BOC) was shown not to be an absolute prerequisite for inhibition, but minor changes of the structure of 2-APB (such as replacement of the boron by a carbon atom and the terminal NH_2 _by a N(CH_3_)_2 _as in diphenhydramine) result in the loss of SOCE inhibitory activity [23]. Diphenylborinic anhydride (DPBA, **2**; Figure 1) and 2,2-diphenyltetrahydrofuran (DPTHF, **3; **Figure 1), two analogues lacking the aminoethyl group were still efficient showing that the aminoethyl group plays no role in SOCE inhibition by 2-APB at least in platelets [23]. More recently, Mikoshiba's group realized the synthesis of 2-APB dimers, some of which demonstrated a 20-45 fold increase in inhibition capacity [25]. However, no structure-function studies have been performed on SOCE potentiation ability of 2-APB analogues.

The aim of this study was to identify which part of the 2-APB molecule is required for potentiation (and inhibition) of SOCE by using analogues modified in the three parts of the molecule: the BOC, the ethanolamine group and the phenyl rings. We show that the BOC is an absolute requirement for the potentiating ability of 2-APB (but not for the inhibitory capacity), and that the number of phenyl rings is linked to the capacity of the molecule to potentiate/inhibit. We also confirm that the ethanolamine group is not required for potentiation and inhibition of SOCE in other cells.

## Results

### Dual effects of borinate esters (2-APB, DPBA and 2-ABB) on SOCE

To visualize the SOCE, we treated the cells with 1 μM thapsigargin (TG) in a Ca^2+^-free medium during 10 min: this treatment allows the release of Ca^2+ ^ions from the ER (Figure 2A), and subsequently the opening of the Store-Operated Channels (SOC). Addition of 1 mM CaCl_2 _then induced an increase of the intracellular Ca^2+ ^concentration ([Ca^2+^]_i_) due to the entry of Ca^2+ ^ions through SOC. As previously described in human Jurkat T [17] and chicken DT40 B cells [18], 2-APB has dual effects on human BL41 B cell SOCE: potentiation at 10 μM (208 ± 8%), inhibition at 50 μM (Figure 2A and 2C). As [Ca^2+^]_i _is the result of an equilibrium between Ca^2+ ^ion influx and Ca^2+ ^efflux, we calculated a Ca^2+ ^ion influx rate when the Ca^2+ ^efflux is greatly reduced [26]. During the first 10-20 s after CaCl_2 _adding, [Ca^2+^]_i _is still low and the Ca^2+ ^pump activity to extrude Ca^2+ ^from the cell is greatly reduced, meaning variations of [Ca^2+^]_i _are due to Ca^2+ ^ion influx. The rate was twofold increased in the presence of 10 μM 2-APB (32.4 ± 7.8 nM/s vs. 62.5 ± 11.5 nM/s). In the presence of 50 μM 2-APB, the Ca^2+ ^influx rate was largely decreased (< 1 nM/s) and the [Ca^2+^]_i _increase after CaCl_2 _adding was largely blunted (Figure 2A).

These results were confirmed by indo-1 quenching experiments with Mn^2+ ^ions: as Mn^2+ ^ions enter the cells by the same channels as Ca^2+ ^ions, they can not be pumped back to the external medium by the plasma membrane Ca^2+ ^ATPases (PMCA) and lead to indo-1 fluorescence quenching at 430 nm [27]. After 10 min treatment by TG to open SOC, addition of 100 μM MnCl_2 _led to a rapid decrease of 430 nm fluorescence (Figure 2B). The quenching was twofold increased in the presence of 10 μM 2-APB (decrease of indo-1 430 nm fluorescence by -0.39 ± 0.03%/s *vs. *-0.22 ± 0.01%/s), but decreased with 50 μM 2-APB (-0.06 ± 0.01%/s *vs. *-0.22 ± 0.01%/s, Figure 2B). In BL41 cells and the absence of TG, CaCl_2 _addition induced a slight increase of [Ca^2+^]_i _(not shown). Thus, these experiments show for the first time that 2-APB directly acts on Ca^2+ ^ion entry through SOC in the human B cell line BL41, with dual effects, potentiation at low, inhibition at higher concentrations.

Diphenylborinic anhydride (DPBA) and 2-aminoethyl dibutylborinate (2-ABB, **4**; Figure 1) have also dual effects on BL41 cell SOCE (Figure 2C). DPBA, a 2-APB analogue with two pairs of diphenylborinate, has recently been shown to inhibit platelet SOCE [23]. In BL41 cells, when DPBA was added, maximal potentiation of SOCE was observed at 3 μM, whereas inhibition was at concentrations higher than 10 μM (Figure 2C). Furthermore, DPBA appeared to potentiate SOCE activity with a slightly greater efficacy than 2-APB (255 *vs. *208%). Thus, BL41 cell SOCE was three times more sensitive to the enhancing and inhibitory effects of DPBA than with 2-APB (Figure 2C). These results were confirmed by indo-1 quenching experiments (Figure 2D): Mn^2+ ^quenching was twofold accelerated with 3 μM DPBA (-0.35 ± 0.02%/s *vs. *-0.17 ± 0.01%/s) and slowed by 50 μM (-0.06 ± 0.02%/s *vs. *-0.17 ± 0.01%/s).

2-ABB, another 2-APB analogue where the two phenyl rings are replaced by two butyl groups, was further shown to have a dual effect on BL41 cell SOCE (Figure 2C), with a maximum of potentiation at 30 μM (201 ± 9%), and inhibition at > 50 μM. Potentiation capability was in the same order of magnitude than 2-APB's (201 *vs. *208%), but slightly weaker than DPBA's (201 *vs. *255%). BL41 SOCE sensitivity to 2-ABB is weaker than for DPBA and 2-APB (Figure 2C). Indo-1 quenching by Mn^2+ ^ions confirmed this result: 30 μM 2-ABB accelerated the quenching (-0.32 ± 0.02%/s *vs. *-0.12 ± 0.01%/s), when 100 μM slowed it (Figure 2E, -0.07 ± 0.02%/s *vs. *-0.12 ± 0.01%/s).

### Differential effects of dimesitylborinic acid (DMBA) and phenylboronic acid (PBA) on SOCE

**Figure 3:**
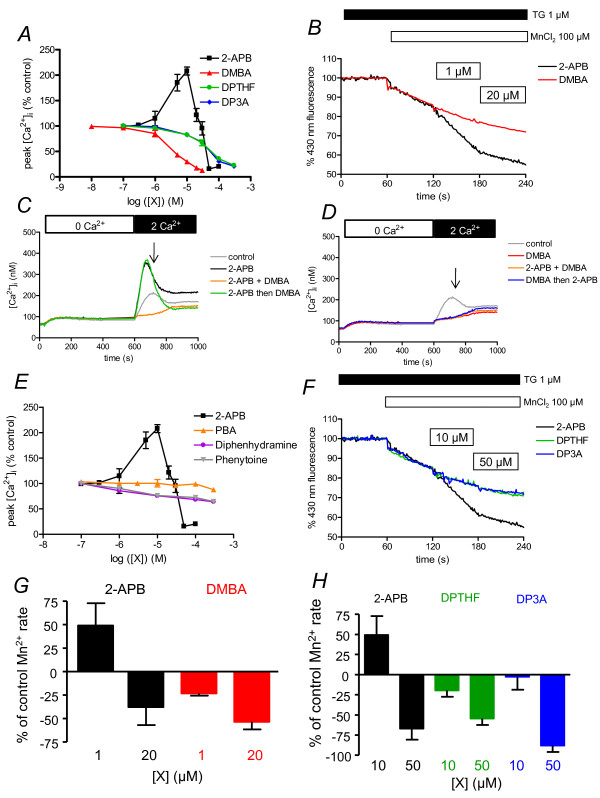
**Non-borinate esters are either uneffective or inhibitors of SOCE**. Dose-response curve of [Ca^2+^]_i _to 2-APB analogues were obtained as in figure 2C. A. DMBA, DPTHF and DP3A are inhibitors of SOCE. B. Mn^2+ ^quenching of indo-1 in BL41 cells stimulated by two concentrations of DMBA or 2-APB. Same as in figure 2B, except that 1 and 20 μM DPBA or 2-APB were used. C and D. Cytosolic calcium concentration measurement of BL41 cells in 2-APB/DMBA competition experiments. Cells were treated 400s with TG 1 μM to allow Ca^2+ ^release from ER and opening of SOC channels. Then 1 mM CaCl_2 _was added, allowing Ca^2+ ^entry through SOC channels. 2-APB 10 μM ("2-APB", C), DMBA 10 μM ("DMBA", D) or 2-APB 10 μM + DMBA 10 μM ("2-APB + DMBA", C and D) were applied 30 s prior to CaCl_2_. In competition experiments, DMBA 10 μM was applied 100 s after CaCl_2 _adding on cells treated by 2-APB ("2-APB then DMBA", C) or 2-APB 10 μM was applied 100 s after CaCl_2 _adding on cells treated by DMBA ("DMBA then 2-APB", D). Error bars were omitted for clarity (maximum ~ ± 50 nM). E. Diphenhydramine and phenytoine are weak inhibitors of SOCE, although PBA is inefficient. Same experiment as done in figure 3A. F. Mn^2+ ^quenching of indo-1 in BL41 cells by low and high concentrations of DPTHF and DP3A, compared to 2-APB. Same protocol was used as in figure 2B. The two analogues were used at 10 μM and 50 μM. G and H. Values of Indo-1 quenching in presence of the borinate esters were normalised to values obtained in the lonely presence of Mn^2+ ^ions, respectively from B and F.

As diphenylborinic acid is unstable, we chose dimesitylborinic acid (DMBA, **5**; Figure 1) which is commercially available and stable. This compound is a 2-APB molecule without an ethanolamine group (and subsequently with a terminal B-OH), and where the two phenyl rings are replaced by two mesityl groups. DMBA was not able to potentiate SOCE at 2-APB potentiating concentrations, but clearly showed an efficient inhibitory capacity (*K*_i _≈ 4 μM, Figure 3A). Indeed, DMBA was close to the efficiency obtained with SKF96365, another common SOCE inhibitor (*K*_i _≈ 3.3 μM, not shown). The inhibitory capacity was confirm by Mn^2+ ^quenching of indo-1: 1 μM DMBA (-0.13 ± 0.01%/s *vs. *-0.17 ± 0.01%/s), 20 μM. (-0.08 ± 0.01%/s *vs. *-0.17 ± 0.01%/s, Figure 3B). As 2-APB and DMBA act in the same range of concentrations, we performed competition experiments. Used at 10 μM, 2-APB was potentiating, DMBA inhibiting (Figure 3C, D). However, when DMBA was added 100 s after CaCl_2_, in the presence of 2-APB (Figure 3C), there was a clear and fast decrease of [Ca^2+^]_i _to values obtained when DMBA is used alone (Figure 3D). Inversely, when 2-APB was added 100 s after CaCl_2 _adding in presence of DMBA (Figure 3D), there was no increase of [Ca^2+^]_i_. Thus, DMBA could impair 2-APB-induced potentiation of SOCE, whereas 2-APB could not avoid DMBA-induced inhibition of SOCE. This shows that DMBA inhibits the SOCE and can bypass the 2-APB-dependent potentiation.

Last we tested phenylboronic acid (PBA, **6**; Figure 1), which can be considered as a hemi-diphenylborinic acid. PBA was devoid of either potentiating or inhibitory activity on SOCE (Figure 3E), suggesting that the two phenyl or mesityl rings must be on the same boron atom to give activity to the 2-APB analogue.

### 2-APB analogues without the B-O core are not able to potentiate SOCE

Thus, compounds containing a terminal B-OH (DMBA and PBA) do not appear to behave like compounds containing a central B-O ("BOC", 2-APB, DPBA and 2-ABB), suggesting a requirement of the BOC for the potentiating activity of 2-APB analogues. To confirm this hypothesis, we performed experiments with 2-APB analogues devoid of B-O. Several of these analogues (Phenytoin, diphenhydramine, DPTHF) have been tested on platelet SOCE and described as inhibitors [23]. It has been hypothesized that the terminal amine and the boron of 2-APB could form an internal coordinate-bound, resulting in the formation of a ring ("close" or "ring", [23]).

Diphenhydramine **(7**; Figure 1), an analogue without boron and where the terminal amine bears two methyl groups cannot form the internal coordinate (analogue of the "open" form of 2-APB). Phenytoine (**8**; Figure 1) is an analogue with a third ring containing two ketone groups and two nitrogen atoms (analogue of the "ring" form of 2-APB). Diphenhydramine and phenytoin were weak inhibitors of BL-41 SOCE (with a maximum of ~35% at 300 μM), and totally devoid of potentiation ability, as previously described on platelets ([23], Figure 3E).

2,2-diphenyltetrahydrofuran (DPTHF (**3**; Figure 1)), an analogue of the 2-APB ring form with a more neutral third ring, was a more effective inhibitor with a *K*_*i *_~ 50 μM, but was totally devoid of potentiating ability (Figure 3A), as in platelets [23]. At 10 μM, DPTHF was a slight inhibitor (-0.11 ± 0.00%/s *vs. *-0.16 ± 0.01%/s), more efficient at 50 μM (-0.06 ± 0.00%/s *vs. *-0.16 ± 0.01%/s) when used in Mn^2+ ^quenching experiments of indo-1 (Figure 3F).

3-(diphenylphosphino)-1-propylamine (DP3A (**9**; Figure 1)) is a 2-APB analogue where the BOC is replaced by a carbon-phosphorus core. This little change resulted in drastic consequences: DP3A was only inhibitory, with a *K*_*i *_similar to DPTHF's (Figure 3A). In indo-1 quenching experiments by Mn^2+ ^ions, DP3A was a weak inhibitor at 10 (-0.11 ± 0.02%/s *vs. *-0.18 ± 0.03%/s) and 50 μM (-0.05 ± 0.02%/s *vs. *-0.18 ± 0.03%/s), confirming its total absence of potentiating effect on the divalent ion influx (Figure 3F). Although DPTHF and DP3A were only inhibitory, they were also less efficient, as higher concentrations were needed to get the same inhibition as done by 2-APB (Figure 3A). This also confirms that the BOC is not an absolute requirement to confer the inhibitory ability of the compound.

### Dual effects of borinate esters (2-APB, DPBA and 2-ABB) are observed in other leukemia/lymphoma cell lines

**Figure 4:**
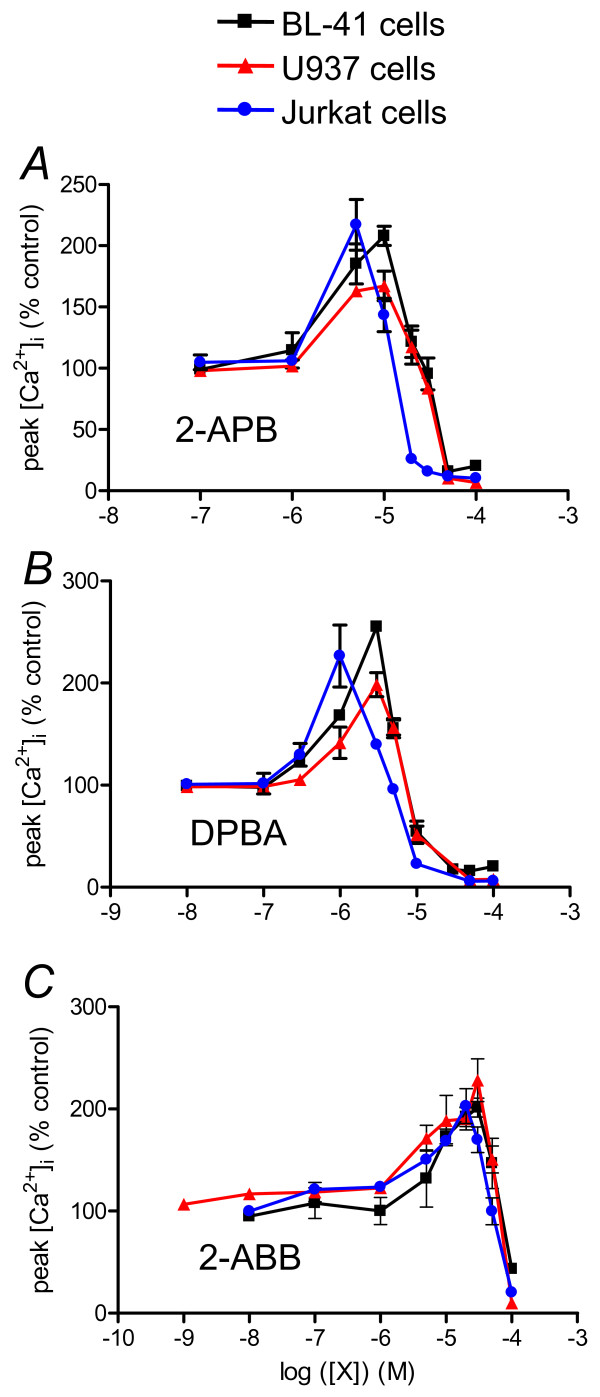
**Borinate esters have dual effects on SOCE of myeloid and lymphoid hematopoietic cells**. Dose response curves for 2-APB (A), DPBA (B) and 2-ABB (C) obtained on SOCE of BL-41, U937 and Jurkat cells. Same protocol was used as in figure 2A to generate the dose-response curve of [Ca^2+^]_i _increase after Ca^2+ ^re-adding to borinate compound concentrations. Values are expressed as % of the peak [Ca^2+^]_i _increase measured in absence of any effector.

As the most extensively studied SOCE is CRAC of T lymphocytes [17] we tested whether the identified structure effects of 2-APB analogues were also relevant to T cells and other cell lines. As previously shown, 2-APB has dual effect on Jurkat cell SOCE, potentiation at 3 μM and inhibition at > 20 μM 2-APB (Figure 4A). SOCE of monocytic U937 cells was potentiated and inhibited by 2-APB in a similar way to BL-41 SOCE: potentiation at 10 μM, inhibition at > 50 μM (Figure 4A). Thus, the 2-APB dose-response curve obtained on Jurkat cell SOCE showed a leftward shift, indicating a near three-fold higher sensitivity to 2-APB than B lymphocyte BL41's and monocytic U937's. Dose-response curves obtained with BL30 (Burkitt lymphoma B cell line), THP1 (monoblastic cell line) and NB4 cells (promyelocytic cell line) were similar to BL41's and U937's (not shown). These results indicate that the previously reported effects of 2-APB on human T cell SOCE [17] can also be observed in other cell types of hematopoietic origin.

DPBA was also 3 fold more efficient than 2-APB in Jurkat and U937 cells (Figure 4B). Remarkably, Jurkat cells remained three times more sensitive to DPBA than the other cell lines, as with 2-APB (Figure 4B).

2-ABB had the same dual effect on U937 and Jurkat cell SOCE (Figure 4C). As with BL41 cells, U937 and Jurkat cells were 2-3 fold less sensitive to 2-ABB than to 2-APB (Figure 4C). Like with 2-APB and DPBA, Jurkat cell SOCE was three-fold more sensitive to the 2-ABB than BL41 and U937 SOCE. Thus, Jurkat cell SOCE is 2-3 fold more sensitive to borinic ester potentiation and inhibition than other cell SOCE.

### Orai isoform expression

**Figure 5:**
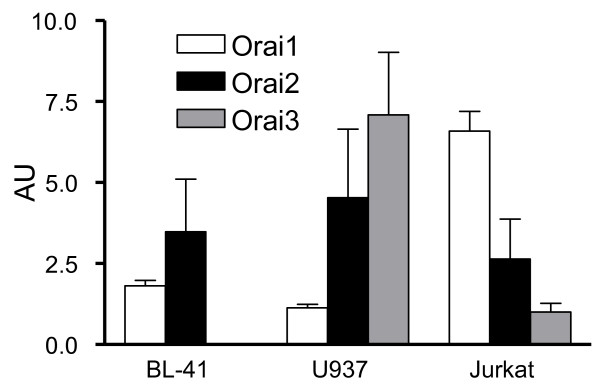
**Orai isoform mRNA are differently expressed in hematopoietic cells**. qRT-PCR results were expressed as arbitrary units with expression of Orai3 in Jurkat equal 1. See material and methods for details.

SOCE is directly dependent on Orai protein expression. Three Orai isoforms have been characterized in human cells and exhibit different sensitivities to 2-APB when over-expressed in HEK 293 cells [28]. We studied Orai isoform expression in our cell lines by using qRT-PCR. As shown in Figure 5, the three cell lines expressed different amounts and ratio of the three isoforms. Thus, although Orai1 is the main isoform in Jurkat T cells at the mRNA scale (with low amount of Orai2 and 3), Orai2 is the main isoform in BL41 B cells and U937 monocytic cells express mainly Orai3, with a slightly weaker amount of Orai2. Noteworthy, BL41 B cells do not express Orai3. Thus, the difference of Orai expression could be responsible of the sensitivity difference to 2-APB/borinate ester dual effects in our cell lines. Even if the Orai1 mRNA is not the main Orais mRNA, the SOCE is almost abolished at [2-APB] > 50 μM in the three cell lines, meaning Orai1 proteins form the main channel.

### Borinate esters are also Ca^2+ ^release inhibitors

**Figure 6:**
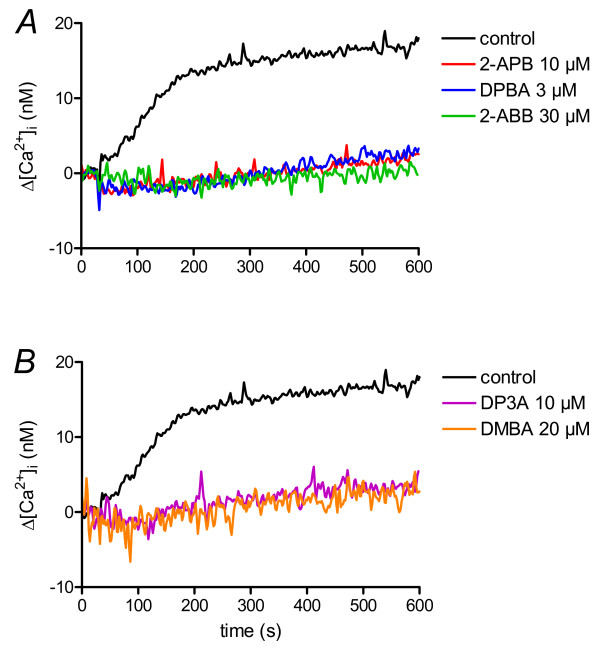
**2-APB and analogues are Ca**^**2+ **^**release inhibitors**. BL-41 cell cytosolic concentrations were measured as in figure 1. To induce Ca^2+ ^release by the ER, cells were treated with 0.5 μg/ml anti-IgM antibody at t = 30 s. According to the treatment, cells were pre-treated with 2-APB or analogues during 5 min prior anti-IgM antibody stimulation. Error bars were omitted for clarity but were < 4 nM.

To test the SOCE-specificity of the 2-APB analogues, we performed the same kind of experiment, except that the cells were stimulated with an anti-IgM antibody instead TG. In these conditions, anti-IgM antibodies induce synthesis of IP_3 _and Ca^2+ ^ion release through IP_3 _receptor of the ER [29]. At their maximal SOCE potentiation concentration, the three borinate esters already impaired the Ca^2+ ^release by the ER (Figure 6A).

DMBA at 10 (not shown) and 20 μM was also able to totally block the Ca^2+ ^release by the ER, in a range of concentration similar to concentration needed to block the SOCE (Figure 6B). In contrast, at a concentration with almost no effect on SOCE (Figure 3A), 10 μM DP3A tightly blocked the Ca^2+ ^release (Figure 6B).

## Discussion

In this work, we clearly show that 2-APB is able to exert dual effects (potentiation and inhibition) on the Ca^2+ ^influx of various cell lines of hematopoietic origin, and may be considered as the parent of a new family of molecules with this dual efficacy. We were able to define which part of the 2-APB molecule is required for potentiation and/or inhibition of the Ca^2+ ^influx through store-operated channels. Despite the same kind of activity in hematopoietic cell lines, a slight difference in sensitivity to 2-APB and its analogues can be observed, and could be due to the relative expression of the three Orai proteins.

Though 2-APB is a small molecule, three parts can be distinguished: an ethanolamine chain, a boron-oxygen core (BOC) and two phenyl rings. The presence of a boron atom with an amine chain allows the formation of an internal coordinate, but the boron atom is also able to interact with N and O atoms of amino-acids, as shown in the case of bortezomib binding to proteasome [30]. The use of analogues helped to understand which part of the 2-APB is important to inhibit SOCE of platelets [22,23], but nothing was known about the potentiation process, as platelet SOCE is clearly not potentiated by 2-APB and analogues.

DPBA had been previously used and was known as a five fold more potent inhibitor of platelet SOCE than 2-APB (2 μM *vs. *10 μM, [23]), meaning the ethanolamine group plays no role in SOCE inhibition (and the N-B internal coordinate bond as well). DPBA could also activate some TRP channels at concentrations > 100 μM [31,32]. In our experiments, DPBA is ~3 fold more efficient than 2-APB, and is able to potentiate the Ca^2+ ^influx. Thus, [Ca^2+^]_i _variations are increased by 200-250% at 3-10 μM, concentrations without any known effect on TRP channels. As DPBA does not possess an ethanolamine group, this confirms that this group plays no role in potentiation process.

2-ABB, a compound never used in Ca^2+ ^signalling, is an analogue of 2-APB where the two phenyl groups were replaced by two butyl. On the three cell lines, we found that 2-ABB, like 2-APB and DPBA, has dual effect on Ca^2+ ^influx, meaning that the two phenyl rings are not an absolute requirement for potentiation and inhibition processes. However, 2-ABB is less potent to potentiate AND inhibit SOCE than 2-APB and DPBA. A clear correlation seems to exist between the number of phenyl rings in the molecule and its ability to potentiate/inhibit SOCE: DPBA (4 phenyl groups) > 2-APB (2) > 2-ABB (0). Thus, it seems that the phenyl rings do not play a direct role in potentiation, but rather favour it. We can assess that after the binding of 2-APB to the channel protein by the BOC, the two phenyl rings strengthen the binding by a second interaction. In DPBA, the presence of four phenyl rings reinforces this binding. It was previously shown that addition of a phenyl ring to the 2-APB molecule could increase its inhibitory efficacy on platelet SOCE [22]. Dimers of 2-APB (with four phenyl rings) had been recently shown to have higher ability to inhibit SOCE than 2-APB [25].

It is also remarkable that the ratio between the concentrations of compound to have maximal potentiation, and to have inhibition is similar with the three borinate esters (from BL41 cells, but same kind from Jurkat and U937 cells): 2-APB 10 μM/100 μM, DPBA 3 μM/30 μM, 2-ABB 30 μM/200 μM (this last value was extrapolated from graphs): this clearly shows that potentiation and inhibition are linked. Interestingly, 2-ABB is the first described SOCE inhibiting molecule without any structural rings.

Sensitivity to potentiation/inhibition by borinate esters varies among cell line SOCE: thus, these compounds are three fold more potent on Jurkat cell SOCE. Using qRT-PCR, we showed the the cell lines used in this study do not express the same Orai proteins, and in different ratios (Figure 5). Thus we confirmed that the main Orai protein expressed by Jurkat T cells is Orai1 [4], although BL41 cells express mainly Orai2, and U937, a mix of Orai2 and Orai3 (Figure 5). Of note, in B cells from patient with a non-functional Orai1 inducing a SCID, SOCE is not abrogated as in T cells, but decreased, allowing a partial NF-AT activation [4,33]. This could be due to the fact that B cells express significantly more Orai2 than Orai1 proteins, and Orai2 proteins could play a role in B cell SOCE. However the role of Orai2 (and Orai3) in SOCE is controversial: although Orai2 (and Orai3) could form SOCE when over-expressed with STIM1 in HEK293 cells [28], heterologous expression of Orai2 in SCID T cells cannot restore SOCE [8]. While Orai1 has been clearly shown to encode the SOC pore in many cells, a recent work showed that Orai3 is the SOC pore of estrogen receptor positive breast cancer cell lines, in contrast to estrogen receptor negative cell line which use Orai1 [34]. The role of transient receptor potential canonical protein (TRPC) in forming store operated channels with Orai1 protein is also controversial: some authors show a clear association [35,36], when others found none [37]. As 2-APB is a known inhibitor of TRPC1, 3 and 6 channels [21], we cannot rule out that in B cells, TRPC proteins form the store-operated channel pore in association with Orai1, and play a role in the SOC pharmacology.

Since the molecular characterization of Orai1 [4], Orai proteins have been fully studied in heterologous expression system [8,28]. Although these systems allow easier characterization, due to larger currents, they should be used with care. Thus, in one of the first studies reconstituting SOCE with Orai1 and STIM1 over-expression in HEK293 cells, it was shown that 2-APB was able to inhibit SOCE but not to enhance it (Figure 1C, [38]). Thus, in over-expressing system with large amount of Orai1 and STIM1 proteins, inducing large increase of [Ca^2+^]_i _(3500 nM), 2-APB is not potentiating. Furthermore, the expression levels of Orai1 and STIM1 have been shown to affect the Ca^2+ ^conductance and the potentiation of the currents by 2-APB [39]. The use of recombinant Orai/STIM seems to be accurate for the study of drugs with inhibition ability, but not for drugs with potentiating ability. Furthermore, SOCE is a large family, with different kinetics and pharmacological properties, and the expression of Orai1 and STIM1 can not reconstitute the different types of SOCE, because all the partners forming a SOC are not known yet (TRPC proteins ? other ?). The use of cell lines to study the pharmacology is complementary to the use of recombinant system, but crucial to identify new compounds with potentiating ability.

The presence of a boron-oxygen link in a molecule does not mean that a compound has potentiating ability on SOCE. DMBA, which resembles 2-APB without the ethanolamine group but a terminal hydroxyl group bound to the boron atom, has shown great capacity to block SOCE, without any potentiating effect. The dipeptidyl boronate pyrazylcarbonyl-Phe-Leu-boronate, also known as PS-341 or bortezomib (commercial name: Velcade^®^) is a well known inhibitor of proteasome used in the treatment of multiple myeloma. Its pharmacophore is a terminal boron atom with two hydroxyl groups [40,41]. Bortezomib blocks protein degradation by binding to the terminal threonine of 20S core particle with a *K*_i _< 1 nM [40,42]. Remarkably, the same molecule without the boron atom ("aldehyde form") largely loses its proteasome inhibitory capacity (*K*_i _= 1.6 μM) [42]. Boron atom presence is also an absolute requirement in serine protease inhibitors [43]. Thus, the same kind of relationship seems to exist between SOC protein/2-APB and proteasome/bortezomib.

The absolute requirement of the boron atom for potentiation was confirmed by DP3A. Despite its closely related structure to 2-APB', DP3A had never been used in Ca^2+ ^signalling before this work. Remarkably, this compound where a P-C core replaces the BOC present in 2-APB, has lost its ability to potentiate SOCE of hematopoietic cell lines (Figure 3A and 3D). This result clearly highlights the key role of the BOC in the potentiation ability of 2-APB. However, DP3A is still inhibitory even if higher concentrations are needed to inhibit SOCE than 2-APB. Thus, replacement of the BOC in the 2-APB molecule impairs the ability of the molecule to potentiate SOCE and decrease its capability to inhibit it. It also confirms that the BOC is not required for a molecule to be inhibitory, as shown previously with DPTHF [23] and in the present work (Figure 3A and 3D).

The dual effect of borinate esters on SOCE is complex to analyse because the binding sites of 2-APB are not clearly established yet. Thus, before the discovery of Orai1, the study of the T cell SOCE, characterized the dual effect of 2-APB and the authors hypothesize the existence of two binding sites, one potentiating with high affinity, the other one with lower affinity and inhibitory [17]. The inhibitory target of 2-APB seems to be STIM1 itself [44], but 2-APB can also bind to the selectivity filter of the Orai protein, widening the size of the pore, resulting in potentiation of the current [45]. However, the same authors provide an alternative explanation where 2-APB interacts with the CRAC channels to widen the channel pore, and when more 2-APB interact with the channel, a change of channel conformation may prevent ions to pass through it. Furthermore, SOC are made with four Orai proteins, meaning a SOC has potentially four 2-APB binding sites, and nothing is known yet about how many 2-APB molecules are required to widen the pore or to block it. Our work could provide new tools to study 2-APB binding sites. Thus, DMBA is a compound able to counteract the potentiating effect of 2-APB. Our preliminary results with DMBA/2-APB competition could not explore the binding sites of the molecules, but we could make two main hypothesis: (i) DMBA and 2-APB have the same binding site. As DMBA impaired 2-APB potentiating effect, and 2-APB can not remove the DMBA inhibition, we could think that DMBA has a higher affinity for the binding site than 2-APB. (ii) DMBA and 2-APB have two different binding sites and binding to the inhibitory binding site (STIM1 ?) bypasses the potentiation binding site. Interesting is the case of Orai3: Orai3 channels do not need stimulation by STIM1 to be opened, and 2-APB has only potentiating ability. If DMBA binds to STIM1, it should have no effect on Orai3 currents. Recently, it has been discovered for the first time that a physiological SOCE could be mediated by Orai3 proteins (usually Orai1' are, [34]). It will be therefore interesting to test the effect of DMBA on these breast cancer cells.

However, all the compounds used in this study are not SOCE specific, as they also exert ability to inhibit the Ca^2+ ^release by the ER. Thus, when borinate esters are at their maximal potentiating concentration, they are already at a concentration fully inhibiting the Ca^2+ ^release (Figure 6), impairing their future use as specific immune-modulator.

## Conclusion

The results from the present study clearly highlight the role of the Boron-Oxygen core in the ability of borinate ester molecules to potentiate Ca^2+ ^entry in hematopoietic cells. The design of potentiating compounds requires the absolute presence of a BOC, whereas design of inhibitors does not. Strikingly, terminal B-OH could give high inhibitory ability to molecules (DMBA). This work could represent the first step for the development of new molecules with immunomodulatory properties: thus, a potentiating compound could be useful to enhance responses of blood cells from immunodeficient patients, or to increase Ca^2+ ^toxicity in leukemia cells.

## Methods

### Cell lines

The BL30 and BL41 (Burkitt B lymphomas), Jurkat (acute T cell leukemia), U937 and THP1 (monocytic cell lines) and NB4 (promyeloid leukaemia cell line) cell lines were maintained in RPMI-1640 medium (Lonza, Verviers, Belgium) supplemented with 10% fetal calf serum and 2 mM ultraglutamine (Invitrogen, Cergy-pontoise, France), at 37°C in a 5% CO_2 _humidified atmosphere. All cell lines used were from ATCC-LGC Promochem (Molsheim, France) except BL-30 and BL-41 cells which were a gift of Irene Joab [46].

### Measurement of intracellular Ca^2+ ^concentration ([Ca^2+^]_i_)

The intracellular Ca^2+ ^concentration was recorded by a fluorimetric ratio technique. Cells were centrifugated and resuspended in Phosphate Buffer Saline medium (PBS, Cambrex, Belgium) supplemented with 1 mg/ml Bovine Serum Albumine (Sigma, Saint Quentin Fallavier, France) under gentle agitation. The fluorescent indicator indo-1 was loaded by incubating the cells at room temperature for 1 h with 4 μM indo-1-AM (Invitrogen/Molecular Probes, Eugene, USA). Cells were then spinned and resuspended in Hepes Buffer Saline (HBS) medium without CaCl_2 _(in mM): 135 NaCl, 5.9 KCl, 1.2 MgCl_2_, Hepes 11.6, glucose 11.5, pH 7.3 adjusted with NaOH.

One million cells were put in a 1 cm width - 3 ml quartz cuvette, and inserted in a spectrofluorophotometer (RF-1501 Shimadzu Corporation, Japan), equipped with a thermostatted (37°C) cuvette holder. Ultraviolet light of 360 nm was used for excitation of indo-1, and emission at 405 and 480 nm were recorded. Background and autofluorescence of the cells were removed from the values measured at 405 and 480 nm. The maximum indo-1 fluorescence (*R*_max_) was obtained by adding 1 μM ionomycin to the bath in the presence of 10 mM CaCl_2_. Minimum fluorescence (*R*_min_) was determined following depletion of external Ca^2+ ^by 5 mM EGTA. All the measures were sent to a PC computer and analyzed. [Ca^2+^]_i _was calculated according to the equation [Ca^2+^]_i _= Kd (R-*R*_min_)/(*R*_max_-R), where *K*d is the apparent dissociation constant of indo-1 for Ca^2+ ^(250 nM, [47]), and R is the ratio of fluorescence value at 405 nM on the one at 480 nm [47].

To induce SOCE, the cells were treated for 10 min with 1 μM TG in the absence of external Ca^2+^. After 10 min, 1 mM CaCl_2 _was added. For dose-response curves, the peak [Ca^2+^]_i _was measured and expressed as a percentage of peak [Ca^2+^]_i _measured in absence of the test compound. These experiments were done at least three times, but for clarity, the SEM was not shown on traces, but is less than 1-2% of the mean.

To calculate the Ca^2+ ^influx rate (Δ[Ca^2+^]_i_), we measure the variation of [Ca^2+^]_i _per second during the first 20 s after CaCl_2 _addition: in these conditions, the activities of the plasma membrane Ca^2+ ^ATPases (PMCA) and of the Na^+^/Ca^2+ ^exchanger are largely reduced (and the activity of SERCA is already inhibited by TG). Thus, the Δ[Ca^2+^]_i _is only due to the Ca^2+ ^ion entry [26].

Mn^2+ ^ion binding to indo-1 forms a non-fluorescent complex. Thus, Mn^2+ ^entry can be estimated by the Indo-1 fluorescence quenching rate [48]. In these experiments, SOCE was activated during 10 min with 1 μM TG without external Ca^2+^. Fluorescence of Indo-1 was recorded at 430 nm emission wavelength (excitation wavelength 360 nm). We recorded this fluorescence during 2-3 min, and only cuvettes with a stable value were then used for the quenching experiment. At t = 60 s, 100 μM MnCl_2 _was added, and the Indo-1 fluorescence quenching was measured, and served as a control value. According to the different experiments, 2-APB or 2-APB analogues were added at various concentrations, and the quenching rates were normalised to the value obtained in presence of only Mn^2+ ^ions. These experiments were done at least three times, but for clarity, the SEM was not shown on traces, but is less than 1-2% of the mean.

To test the effects of 2-APB and its analogues on the Ca^2+ ^release by the ER, the BL41 cells were stimulated by an anti-IgM antibody (ABCam, Paris, France) instead by TG, still in absence of extracellular Ca^2+^. This kind of antibody cross-links to the B cell receptor induces the synthesis of IP_3 _and Ca^2+ ^release through IP_3 _receptors [29]. Cells were pretreated 5 min with 2-APB or an analogue, then stimulated by 0.5 μg/ml anti-IgM antibody. These experiments were done at least three times, but for clarity, the SEM was not shown on traces, but is less than 1-2% of the mean.

### Orai isoform expression

Total RNA was isolated from cell lines by using the TRIzol^® ^Reagent (Invitrogen) following the manufacturers' instructions. RNA was quantified with Thermo Scientific NanoDrop™ Spectrophotometers. Reverse transcription was performed on 1 μg of total RNA. After 3 min at 95°C, RNA was incubated for 1 h at 42°C with 50 units of M-MLV reverse transcriptase (Applied Biosystems), 20 units of RNase inhibitor (Applied Biosystems), 2.5 mM random hexamers and dNTP at 1 mM in a final volume of 20 μl. Finally, reverse transcriptase was denatured by heating for 5 min at 95°C. We quantified transcripts of the *GAPDH *gene as the endogenous RNA control. Each sample was normalized on the basis of its *GAPDH *content, and results expressed as *N*-fold differences in target gene expression relative to the *GAPDH *gene (termed *N*target), were determined with the following formula: *Ntarget *= *2*(Δ*Ct sample *- Δ*Ct calibrator*), where the ΔCt value of the sample was determined by subtracting the average Ct value of the target gene from the average Ct value of the *GAPDH *gene. The *N*target values of the samples were subsequently normalized such that the mean ratio of the samples with the lowest expression level would have a value of 1. The nucleotide sequences of the primers used for PCR amplification were as follows:

GAPDH-Forward: GGACCTGACCTGCCGTCTAGAA

GAPDH-Reverse: GGTGTCGCTGTTGAAGTCAGAG

ORAI1-Forward: TCGGTCAAGGAGTCCCCCCAT

ORAI1-Reverse: GTCCTGTAAGCGGGCAAACTC

ORAI2-Forward: GCTGAGCTTAACGTGCCTATC

ORAI2-Reverse: GGAGTTCAGGTTGTGGATGTT

ORAI3-Forward: CCCTTAGTCCAGCTTCCAATC

ORAI3-Reverse: CCAAGGAGCGGTAGAAATGCA

PCR was performed using an ABI Prism 7700 Sequence Detection system (Perkin-Elmer Applied Biosystems) and the SYBR^® ^Green PCR Master Mix (Applied Biosystems). The thermal cycling conditions comprised an initial denaturation step at 95°C for 10 min and 40 cycles at 95°C for 15 s and 60°C for 1 min. Experiments were performed in duplicates for each data point.

### Chemicals

2-aminoethoxydiphenyl borate (2-APB), 2-aminoethoxydibutyl borate (2-ABB), diphenylboronic anhydride (DPBA), 2,2-diphenyltetrahydrofuran (DPTHF), phenylborinic acid (PBA), 3-(diphenylphosphino)-1-propylamine (DP3A), phenytoin, diphenhydramine, ,dimesitylborinic acid (DMBA) and 2-aminoethyl dibutylborinate (2-ABB) were from Sigma-Aldrich (Saint Quentin Fallavier, France), thapsigargin was purchased from Calbiochem-Merck (Nottingham, UK). The structure of 2-APB and analogues is given in Figure 1.

### Statistical analysis

Given values are representative of at least 3 independent experiments ± SEM. When used, a t-test < 0.05 is considered as significant.

## List of abbreviations

SOCE: Store-operated calcium entry; SOC: Store-Operated Channel; CRAC: Calcium-Release Activated Calcium; IP_3_: inositol 1,4,5-trisphosphate; 2-APB: 2-aminoethoxydiphenylborate (2-aminoethyl diphenylborinate); [Ca^2+^]_i_: intracellular Ca^2+ ^concentration; ER: endoplasmic reticulum; TRP: transient receptor potential; BOC: boron-oxygen core; TG: thapsigargin; DPBA: diphenylborinic anhydride; DPTHF: 2,2-diphenyltetrahydrofuran; PBA: phenylborinic acid; DP3A: 3-(diphenylphosphino)-1-propylamine; DMBA: dimesitylborinic acid; 2-ABB: 2-aminoethyl dibutylborinate.

## Authors' contributions

OD carried out the [Ca^2+^]_i _measurements and Orais expression assays, and drafted the manuscript. PM carried out some [Ca^2+^]_i _measurements and compound tests.

CC conceived and designed the study. All authors have approved the final manuscript
